# Contribution of Signal Transducer and Activator of Transcription 3 (STAT3) to Bone Development and Repair

**DOI:** 10.3390/ijms25010389

**Published:** 2023-12-27

**Authors:** Mohamed L. Sobah, Clifford Liongue, Alister C. Ward

**Affiliations:** 1 School of Medicine, Deakin University, Waurn Ponds, Geelong, VIC 3216, Australia; mlsobah@deakin.edu.au; 2 Institute of Mental and Physical Health and Clinical Translation (IMPACT), Deakin University, Waurn Ponds, Geelong, VIC 3216, Australia; c.liongue@deakin.edu.au

**Keywords:** STAT3, bone, regeneration, macrophages, neutrophils

## Abstract

Signal transducer and activator of transcription 3 (STAT3) is a transcription factor activated canonically by numerous cytokines and other factors, with significant roles in immunity, immune diseases, and cancer. It has also been implicated in several human skeletal disorders, with loss-of-function (LOF) mutations associated with aberrant skeletal development. To gain further insights, two zebrafish STAT3 lines were investigated: a complete LOF knockout (KO) mutant and a partial LOF mutant with the transactivation domain truncated (ΔTAD). Consistent with other studies, the KO mutants were smaller, with reduced length in early embryos exacerbated by a decreased growth rate from 5 days postfertilization (dpf). They displayed skeletal deformities that approached 80% incidence by 30 dpf, with a significant reduction in early bone but not cartilage formation. Further analysis additionally identified considerable abrogation of caudal fin regeneration, concomitant with a paucity of infiltrating macrophages and neutrophils, which may be responsible for this. Most of these phenotypes were also observed in the ΔTAD mutants, indicating that loss of canonical STAT3 signaling was the likely cause. However, the impacts on early bone formation and regeneration were muted in the ΔTAD mutant, suggesting the potential involvement of noncanonical functions in these processes.

## 1. Introduction

Signal transducer and activation of transcription 3 (STAT3) is best known as a transcription factor canonically activated downstream of multiple cytokines involved in immune system development and function [1]. However, STAT3 has also been established as a crucial regulator of bone development and homeostasis, with a number of key roles identified in the formation and maintenance of both osteoblasts and osteoclasts [2]. Here, it acts downstream of multiple cytokines and other factors, including interleukin 6 (IL-6) family members, such as leptin [3], IL-6 and leukemia inhibitory factor (LIF) [4], as well as IL-17A [5], myeloid-derived growth factor (MYDGF) [6], and HIF-1A [7]. The effects of STAT3 are mediated through its activation of critical genes involved in osteoblast and osteoclast differentiation, including the cytokine RANKL [2] and the transcription factors NFATc1 [8] and RUNX2 [2]. Moreover, STAT3 can participate in noncanonical modes of action [9] that may also be important.

As global knockout of STAT3 in mice is embryonic lethal [10], conditional knockouts have been developed to study its roles in specific cell lineages. Osteoblast-specific STAT3 ablation resulted in decreased bone formation, craniofacial deformities, dwarfism, and osteoporosis, with heterozygous knockout mice also displaying a skeletal phenotype with osteoporosis and low mineral bone density [11]. Similar skeletal phenotypes with low bone density have been observed following STAT3 ablation in hematopoietic stem cells [12] or chondrocytes [4], or alternatively following chondrocyte-specific ablation of GP130, the common signaling chain for the IL-6 family of cytokines [4].

STAT3 has also been implicated in several human bone-related disorders [13]. Notably, germline loss-of-function (LOF) STAT3 mutations represent the most common cause of autosomal dominant hyper IgE syndrome (AD-HIES) [14]. AD-HIES is a complex disorder that includes several immunological presentations, along with skeletal, dental, and/or connective tissue defects [15,16,17]. The skeletal deformities present as distinct facial and dental features, scoliosis, and hyperextensibility of joints [15]. Alternatively, increased activation of STAT3 has been implicated in other bone-related disorders such as osteoporosis and osteoarthritis (OA) [18]. In a mouse model of OA, Stat3 activation downstream of IL-6 was associated with cartilage destruction, which could be ameliorated with a STAT inhibitor [19]. Therefore, better understanding of how Stat3 impacts bone and other skeletal elements will aid with the development of therapies for relevant diseases.

Zebrafish is a well-established model for the study of skeletal disorders. Cell lineages and cellular markers for osteoblasts and osteoclasts as well as their precursors are largely conserved in zebrafish, while their transparency and regenerative capabilities provides new avenues for research [20,21]. The pathways involving Stat3 are also conserved in zebrafish, with the zebrafish Stat3 protein retaining all functional domains and showing strong sequence homology [22], with good conservation of upstream and downstream signaling components [23,24]. Zebrafish *stat3* is also expressed in similar cell lineages to those in mammals and is activated downstream of conserved pathways [22,25,26,27]. Unlike mouse models, zebrafish Stat3 knockouts were able to undergo successful embryogenesis but exhibit scoliosis and growth defects, succumbing to early lethality during their late larval stage [25,26]. Here, we utilized two newly developed zebrafish Stat3 mutant lines to further understand the impacts on skeletal development and repair, including the involvement of macrophages and neutrophils, and consider the potential for noncanonical functionality.

## 2. Results

### 2.1. Stat3 Mutations Affect Development

**Figure 1:**
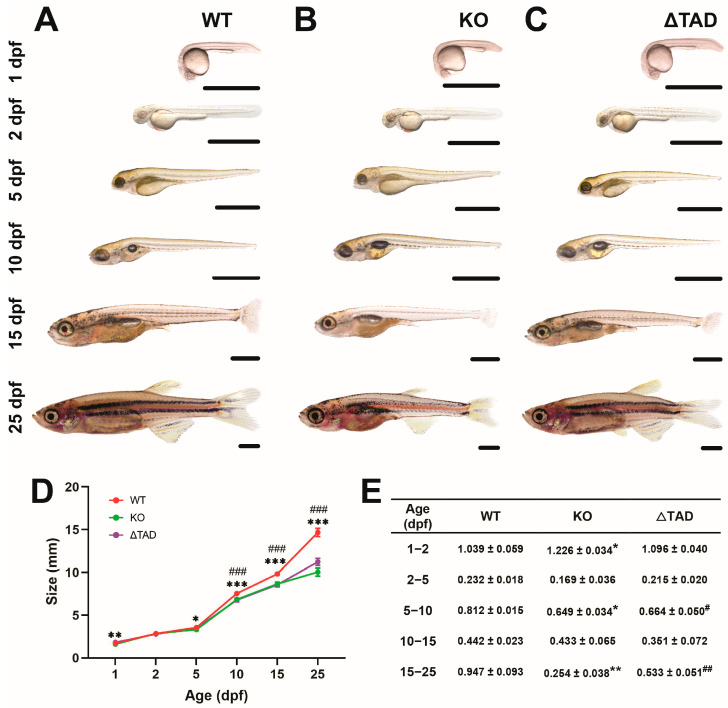
Effect of Stat3 mutations on size and growth. Representative images of Stat3 wildtype (WT: **A**), knockout (KO: **B**), and transactivation domain truncation (ΔTAD: **C**) mutants at the indicated times postfertilization with quantitation of length (**D**) and calculated growth rate (**E**) at the given time, presented as mean with the standard error of the mean (SEM) and statistical significance indicated between WT and KO (*** *p* < 0.001, ** *p* < 0.01, * *p* < 0.05) as well as WT and ΔTAD (### *p* < 0.001, ## *p* < 0.01, # *p* < 0.05).

We have recently generated a Stat3 knockout (KO) zebrafish mutant, containing a frameshift and early stop codon in the N-terminal domain leading to a severely truncated protein, as well as a mutant with a frameshift and early stop codon toward the end of the SH2 domain resulting in complete transactivation domain truncation (ΔTAD) (Sobah, M.L., et al., 2024 [28]). Homozygous Stat3 KO and ΔTAD mutants were both noticeably smaller than their wildtype (WT) counterparts (Figure 1A–C). Stat3 KO zebrafish mutants were already significantly shorter at 1 day postfertilization (1 dpf), which continued and became exacerbated at 5 dpf, 10 dpf, 15 dpf, and 25 dpf (Figure 1D). The calculated growth rates were significantly lower than the WT equivalents between 1–2 dpf, 5–10 dpf, and 15–25 dpf (Figure 1E). Similarly, Stat3 ΔTAD mutants were significantly smaller than their WT equivalents at 5 dpf, 10 dpf, 15 dpf, and 25 dpf (Figure 1D), with the growth rate also significantly reduced between 5–10 dpf and 15–25 dpf (Figure 1E).

**Figure 2:**
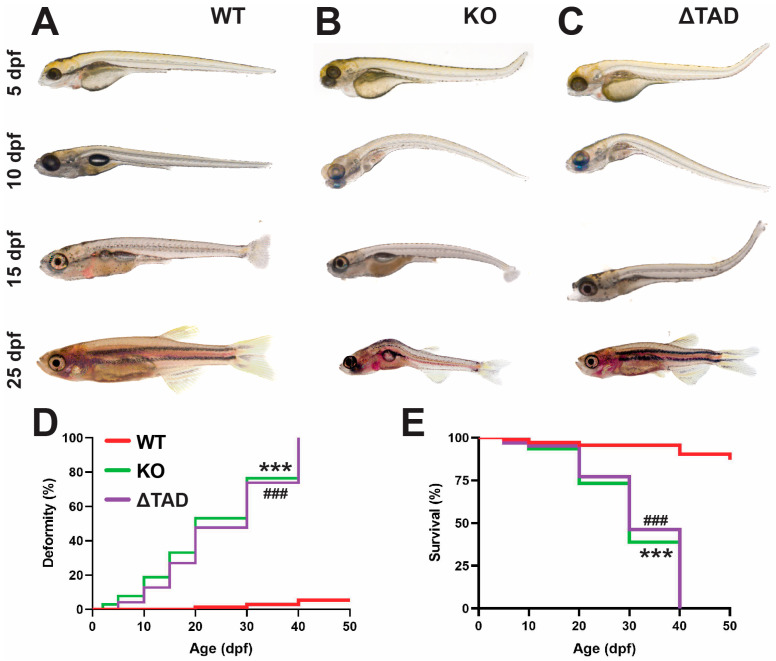
Deformity and mortality of Stat3 mutants. Representative images of Stat3 wildtype (WT: **A**), knockout (KO: **B**), and transactivation domain truncation (ΔTAD: **C**) mutants presenting with deformities at the indicated time points. The incidence of deformity (**D**) and relative survival (**E**) in these mutants compared to wildtype (WT) individuals are presented as Kaplan–Meier plots, with statistical significance indicated between WT and KO (*** *p* < 0.001) as well as WT and ΔTAD (### *p* < 0.001).

Both Stat3 mutants additionally exhibited significant deformities from early larval stages, not evident in Stat3 WT (Figure 2A). For the Stat3 KO mutants, deformed embryos were observed as early as 2 dpf, with bending of the spine clearly evident from 5 dpf (Figure 2B) and jaw defects at 15 dpf (Supplementary Figure S1B,D) but not in Stat3 WT (Supplementary Figure S1A,D), with the proportion of deformed fish increasing steadily, reaching 100% by 40 dpf (Figure 2D). Stat3 ΔTAD mutants also developed similar, albeit slightly less severe, deformities at an equivalent rate (Figure 2D, Supplementary Figure S1C,D). Both mutants also succumbed to early lethality that largely mirrored the onset of deformity (Figure 2E).

**Figure 3:**
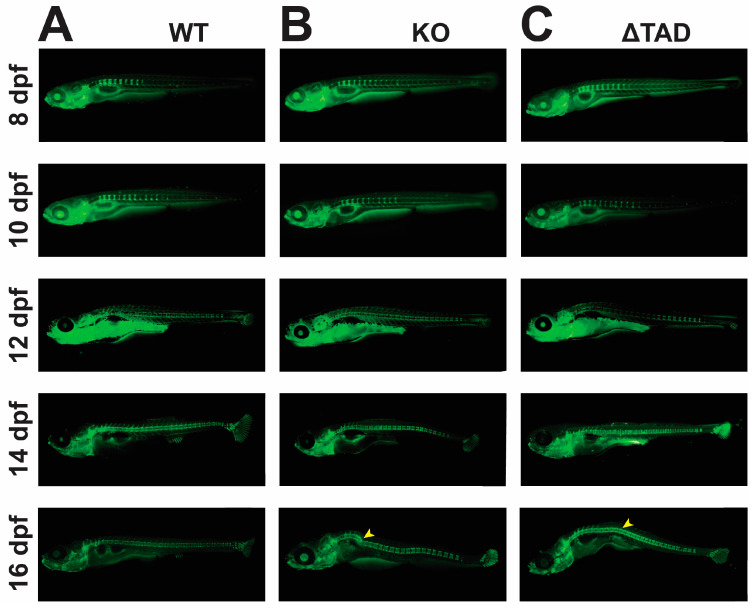
Effects of Stat3 mutants on spine formation. Representative images of calcein-stained Stat3 wildtype (WT: **A**), knockout (KO: **B**), and transactivation domain truncation (ΔTAD: **C**) mutants at the indicated timepoints, with yellow arrowheads indicating skeletal disruption.

Spinal development of the Stat3 mutants was also assessed through calcein green staining. During early spinal development at 8–10 dpf, no obvious differences in spine formation were evident between the two Stat3 mutants and their WT counterparts, with a comparable number of vertebrae present (Figure 3A–C). After this time, the Stat3 mutants started to exhibit spinal deformity, clearly visible from 16 dpf as bends in the spinal column that were more severe in the KO, although again there was a comparable vertebrae number in both mutants compared to those of WT larvae (Figure 3A–C).

### 2.2. Stat3 Is Required for Bone Development

**Figure 4:**
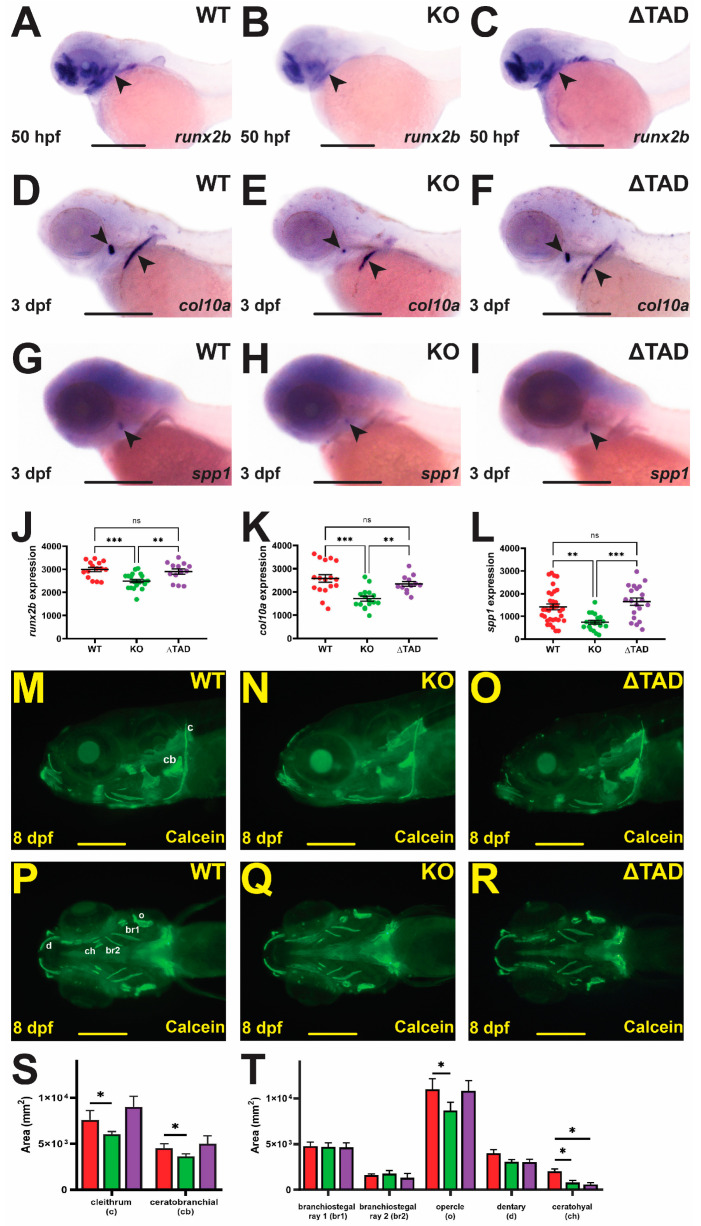
Effect of Stat3 mutations on bone formation. Representative images of Stat3 wildtype (WT: **A**,**D**,**G**,**M**,**P**), knockout (KO: **B**,**E**,**H**,**N**,**Q**), and transactivation domain truncation (ΔTAD: **C**,**F**,**I**,**O**,**R**) mutant embryos subjected to WISH at 50 hpf with *runx2b* (**A**–**C**) and at 3 dpf with *col10a* (**D**–**F**) or *spp1* (**G**–**I**) in lateral view as indicated with black arrowheads, or at 8 dpf with calcein staining (lateral: **M**–**O**; ventral: **P**–**R**), with scale bars representing 200 μm. Quantification of area of staining for *runx2b* (**J**), *col10a* (**K**), and *spp1* (**L**), showing individual values for each embryo and of specific bones (**S**,**T**), with mean, SEM, and statistical significance indicated (*** *p* < 0.001, ** *p* < 0.01, * *p* < 0.05, ns: not significant). Abbreviations: br1/2: branchiostegal ray 1/2; c: cleithrum; cb: ceratobranchial; ch: ceratohyal; d: dentary; o: opercula.

To test the effect of Stat3 mutations on bone development, expression analysis was performed using whole-mount in situ hybridization (WISH) with specific gene markers. Expression of the pre-osteoblast marker *runx2b* [29] was significantly diminished in Stat3 KO embryos at 50 hpf (Figure 4B,J). Similarly, expression of the osteoblast markers *col10a* [30] (Figure 4E,K) and *spp1* (osteopontin) [31] (Figure 4H,L) were also significantly decreased in Stat3 KO compared to WT embryos. In contrast, no significant changes in the expressions of *runx2b* (Figure 4C,J), *col10a* (Figure 4F,K), or *spp1* (Figure 4I,L) were observed in Stat3 ΔTAD embryos. Bone formation was visualized with calcein staining, which revealed significant decreases in the cleithrum, ceratobranchial bone, opercle, and ceratohyal bone in Stat3 KO larvae at 8 dpf (Figure 4N,Q,S,T). In comparison, the Stat3 ΔTAD mutants showed normal development of all bones except for the ceratohyal bone, which was significantly smaller compared to that of WT larvae (Figure 4O,R–T).

### 2.3. Stat3 Is Dispensable for Cartilage Formation

**Figure 5:**
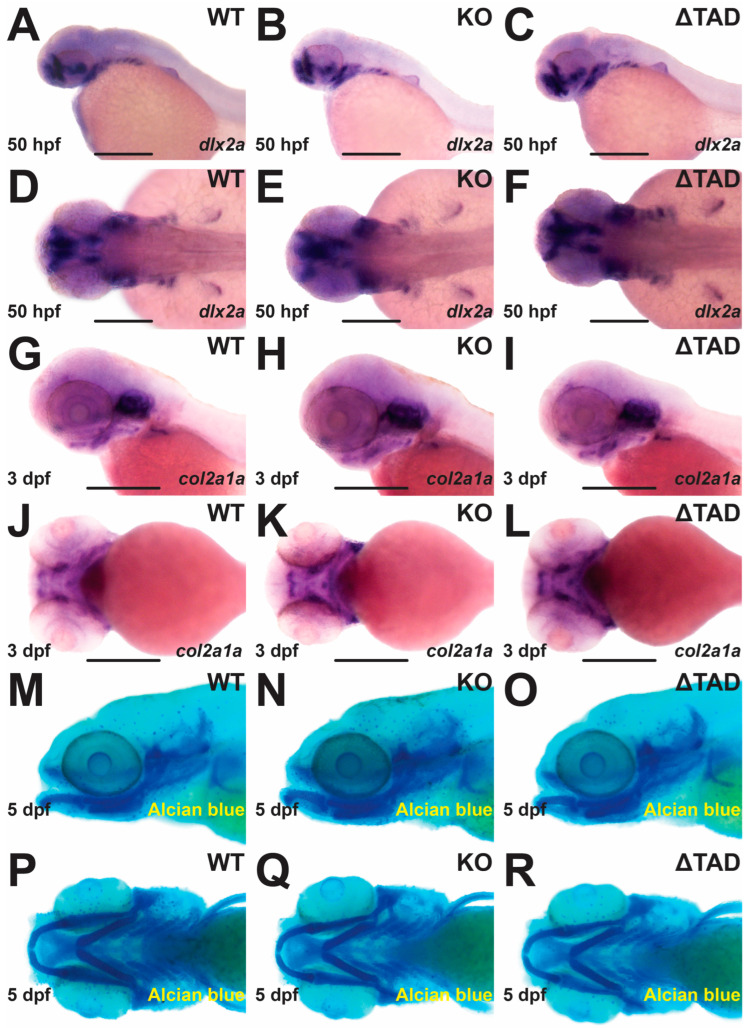
Effect of Stat3 mutations on embryonic cartilage formation. Representative images of STAT3 wildtype (WT: **A**,**D**,**G**,**J**,**M**,**P**), knockout (KO: **B**,**E**,**H**,**K**,**N**,**Q**), and transactivation domain truncation (ΔTAD: **C**,**F**,**I**,**L**,**O**,**R**) mutant embryos subjected to WISH at 50 hpf with *dlx2a* (lateral: **A**–**C**; ventral: **D**–**F**) and at 72 hpf with *col2a1a* (lateral: **G**–**I**; ventral: **J**–**L**), or at 5 dpf to Alcian blue staining (lateral: **M**–**O**; ventral: **P**–**R**) with scale bars representing 200 μm.

To investigate the effect of Stat3 mutations on cartilage formation, WISH with specific gene markers was also employed. No differences were observed between either Stat3 KO or ΔTAD mutants and WT embryos using either of the chondrocyte markers *dlx2a* [32] (Figure 5A–F) or *col2a1a* [33] (Figure 5G–L). Cartilage formation was also visualized through Alcian blue staining, which revealed both Stat3 KO and ΔTAD mutants had equivalent cartilage to their WT counterparts at 5 dpf (Figure 5M–R).

### 2.4. Stat3 Is Essential for Fin Ray Regeneration and the Associated Innate Immune Response

**Figure 6:**
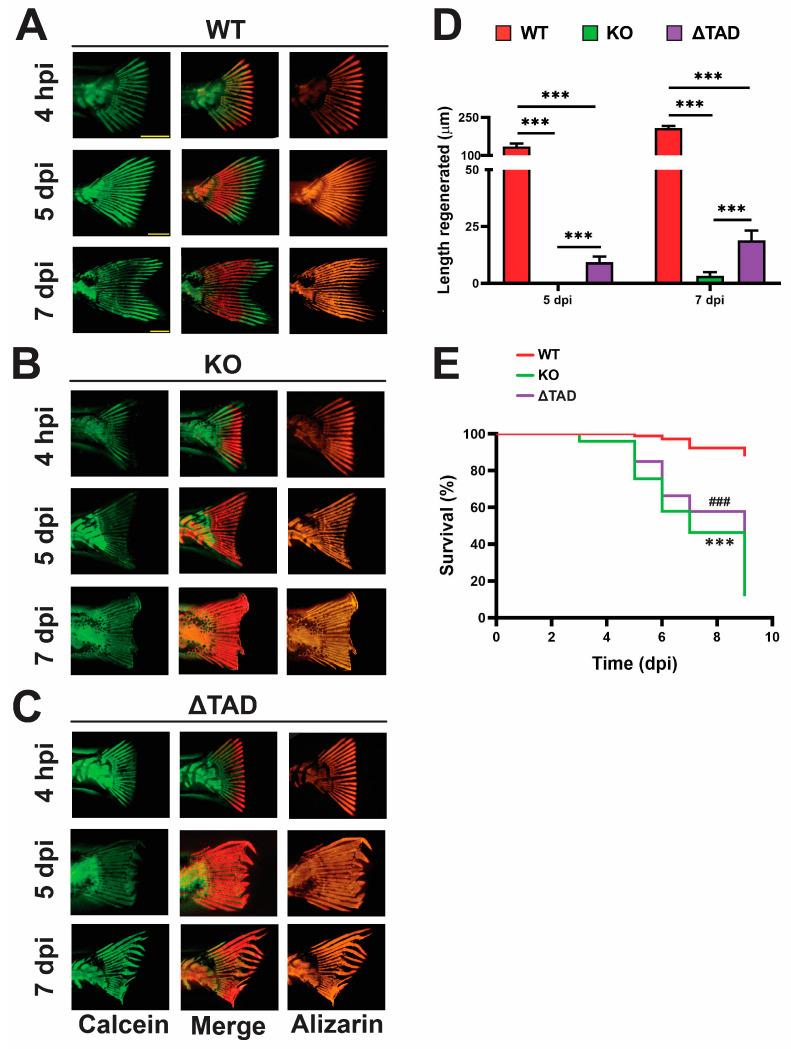
Effect of Stat3 mutations on fin regeneration. Representative images of wildtype (WT: **A**), knockout (KO: **B**), and transactivation domain truncation (ΔTAD: **C**) zebrafish subjected to staining with calcein and Alizarin red at the indicated timepoints, showing staining with each separately and then overlaid (Merge), with scale bars representing 1 mm. Quantification of caudal fin regeneration as the length from the amputation site to the newly formed fin ray tip (**D**) showing mean with SEM and statistical significance indicated (*** *p* < 0.001). Relative survival following amputation (**E**), presented as a Kaplan–Meier plot with statistical significance indicated between WT and KO (*** *p* < 0.001) as well as WT and ΔTAD (### *p* < 0.001).

Regeneration was evaluated in 15 dpf zebrafish larvae subjected to amputation of the caudal fin. At 4 h postinjury (hpi) as well as 5 and 7 days postinjury (dpi), larvae were subjected to double calcein green/Alizarin red staining to identify newly calcified bone, which readily stains with calcein as opposed to previously deposited bone matrices staining preferentially with Alizarin red [20], with the extent of regeneration quantified at 5 and 7 dpi. WT fish showed robust regeneration (Figure 6A,D) and very good survival postamputation (Figure 6E). In contrast, negligible regeneration was observed in the Stat3 KO zebrafish at 5 dpi, with the amputation site appearing very similar to those at 4 hpi and only minor regeneration by 7 dpi (Figure 6B,D). Stat3 ΔTAD mutants also showed significantly reduced regeneration compared to the WT at both time points; however, the length regenerated was significantly higher than that of Stat3 KO mutants at the same time point (Figure 6C,D). Furthermore, both Stat3 KO and Stat3 ΔTAD mutants exhibited deformed regeneration relative to the WT, with fin bone matrices deposited in a disordered manner, impacting the fin pattern (Supplementary Figure S2A–C). This was quantified by measuring the angular deviation between the original and newly formed fin ray, for which we identified a significant decrease in both Stat3 KO and Stat3 ΔTAD mutants compared to the WT (Supplementary Figure S2D). Lastly, both mutants also showed similar statistically significant decreases in survival following amputation compared to their WT counterparts (Figure 6E).

**Figure 7:**
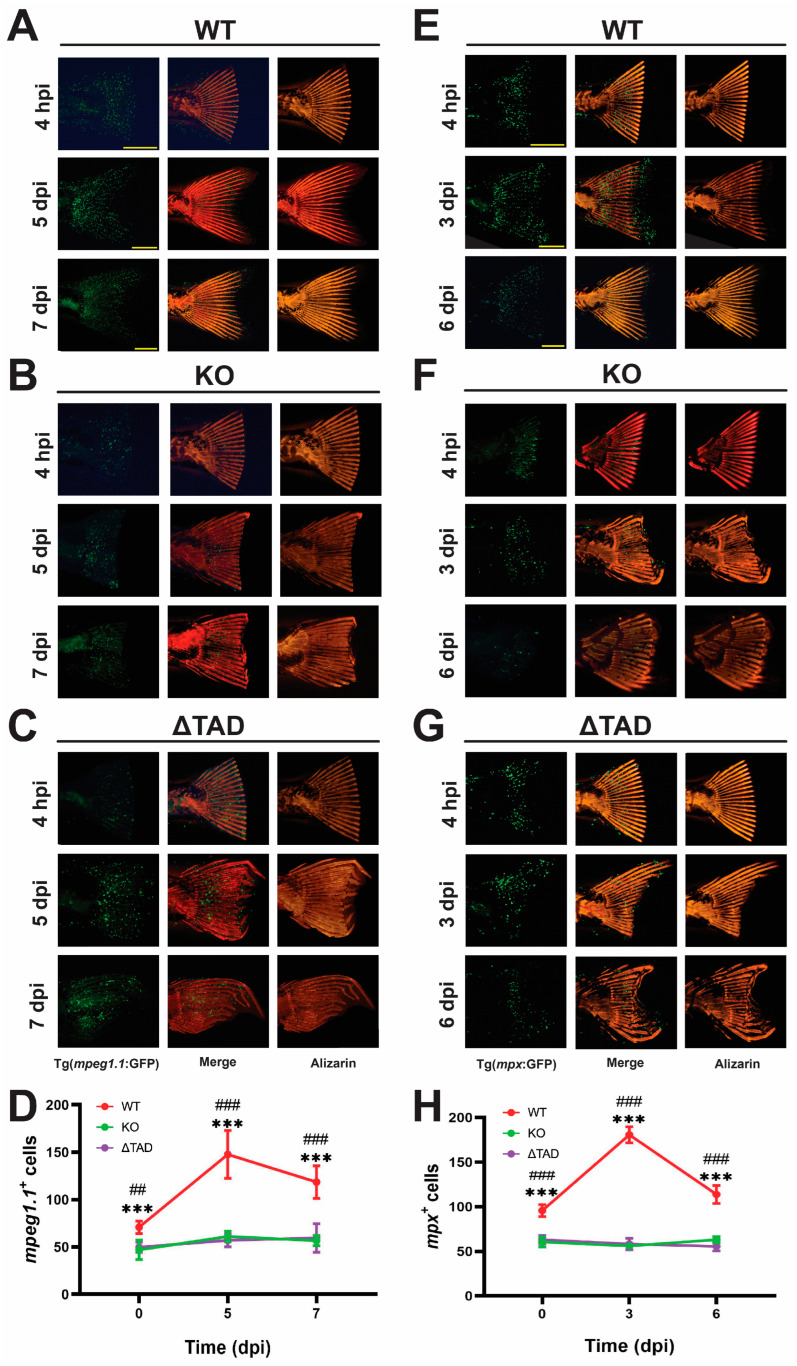
Effect of Stat3 mutations on innate immune response to amputation. Representative caudal fin of wildtype (WT: **A**,**E**), knockout (KO: **B**,**F**), and transactivation domain truncation (ΔTAD: **C**,**G**) zebrafish larvae on either the Tg(*mpeg1.1*:GFP) (**A**–**C**) or Tg(*mpx*:GFP) (**E**–**G**) backgrounds stained with Alizarin red (Alizarin) at the timepoints indicated, showing GFP fluoresce and Alizarin staining separately and then overlaid (Merge), with scale bars representing 1 mm. Quantitation of *mpeg1.1*+ cells (**D**) and *mpx*+ cells (**H**) at the indicated times postamputation with mean and SEM, showing statistical significance between WT and KO (*** *p* < 0.001) as well as WT and ΔTAD (### *p* < 0.001, ## *p* < 0.01).

To assess the impacts on macrophages and neutrophils following amputation, Stat3 mutants on the Tg(*mpeg1.1*:GFP) and Tg(*mpx*:GFP) transgenic backgrounds, respectively, were used. The caudal fins of these were amputated and imaged over time using Alizarin red to visualize the skeletal structure, and GFP was used to visualize and quantify macrophages and neutrophils, as previously described [34]. Macrophage numbers in the caudal fin of the WT zebrafish significantly increased at 5 dpi and then decreased at 7 dpi (Figure 7A,D). In contrast, there was no significant change in the number of macrophages in Stat3 KO (Figure 7B,D) or Stat3 ΔTAD (Figure 7C,D) mutants, for which both were significantly decreased compared to that of the WT at all time points (Figure 7D). Neutrophil numbers were similarly increased in WT zebrafish with a peak at 5 dpi (Figure 7E,H), while no such increase was observed in either Stat3 KO (Figure 7F,H) or Stat3 ΔTAD (Figure 7G,H) mutants, with both again significantly lower than that of the WT at all time points (Figure 7H).

## 3. Discussion

STAT3 has been widely studied in the context of immune diseases and cancer [1,13,35]. However, more recently, it has garnered significant attention for the significant role it plays in various skeletal disorders, including osteoporosis [2] and the skeletal deformities associated with AD-HIES [16]. Mouse models have provided significant insight onto the functions of STAT3 in skeletal development and its perturbation in disease but have typically relied on lineage-specific ablation of STAT3 and other pathway components [2,8,11,36]. Previous studies on other zebrafish Stat3 KO lines have revealed growth defects and skeletal deformities, with bone but not cartilage affected [25,26,37]. They also identified an early convergence-extension defect that impacts the initial embryo length [26] and joint fractures that likely contribute to the spinal curvature, with evidence of inflammation at later stages [25]. To further explore the impacts of STAT3 on bone, cartilage, and skeletal formation, we employed our recently generated Stat3 KO zebrafish, and we additionally studied the impacts on fin regeneration following injury that included analysis of innate immune system components. We also examined in parallel the alternative Stat3 ΔTAD mutant to allow potential noncanonical functions to be considered.

The Stat3 KO mutants displayed significantly reduced *stat3* expression (Supplementary Figure S3) and similar defects in growth and skeletal development as observed with the other zebrafish Stat3 KO mutants, which together provide support that this reflects a hypomorphic mutant. Bone formation was explored through analysis of dermal bones as key indicators of bone development in zebrafish [38,39,40]. The cleithrum, operculum, ceratobranchial, and ceratohyal bones were all significantly smaller in Stat3 KO mutants, along with reduced expression of the key osteoblast markers *runx2b*, *col10a*, and *spp1*. Stat3-mediated *Runx2* expression has separately been shown to be important during in vitro mouse osteoblast differentiation [41], as well bone formation and responses to stress via mechanical stretching [42]. Stat3 ablation in mouse osteoblasts also resulted in disrupted bone development, with stunted growth and reduced bone density attributed to decreased expression of *Dlx5* [11]. This gene is required for osteoblast maturation and function, with *Dlx5^−/−^* mice showing similar skeletal features to Stat3-deficient mice [43]. Expression of the zebrafish *Dlx5* orthologues, *dlx5a* and *dlx5b*, was observed in early skeletal landmarks, with knockdown of *dlx5a* resulting in a similar axial elongation defect as that in the Stat3 KO mutant, as well as decreased expression of both *runx2b* and *col10a* [44]. Analysis of Stat3 KO mutants via qRT^2^-PCR also revealed a significant decrease in *dlx5a* expression. Collectively, this suggests a conserved Stat3/Dlx5/Runx2/Col10 pathway is crucial in early bone development. In contrast, ablation of Stat3 in mouse osteoclasts resulted in increased bone mass [8].

In contrast, early cartilage formation was not significantly impacted in Stat3 KO mutants, evidenced through Alcian blue staining and analysis of the cartilage markers *col2a1a* and *dlx2a*, and confirmed via qRT^2^-PCR for the additional markers *sox9a* and *sox9b*. These results were contradictory to some mouse studies. For example, Stat3 ablation in chondrocytes significantly impaired cartilage formation [4]. Meanwhile, ablation of Stat3 in mesoderm cells resulted in spinal curvature and dysregulated endochondral ossification [45]. However, ablation of Stat3 in mouse osteoprogenitor cells resulted in defective osteoblast development and skeletal deformities, but normal chondrocyte differentiation and cartilage formation [36], indicating cartilage disruption is not a prerequisite for skeletal defects.

Fin regeneration was almost completely absent in the Stat3 KO mutants, along with innate immune cell infiltration to the injury site. Macrophages have been shown to be critical for regeneration in multiple contexts in zebrafish, including axonal, cardiac, and caudal fins [46,47], but also following fractures in mice [48]. In caudal fin regeneration, macrophages play a key role in the resolution of the inflammatory response that is otherwise inhibitory [49]. Therefore, the lack of macrophage infiltration likely contributed to the defective regeneration observed. However, the extent of the regeneration defect was greater than that reported in the absence of macrophages [34], suggesting other contributions. Of note, neutrophils infiltration was also absent. Neutrophils are typically among the first cells to respond to injury, promoting wound healing via the secretion of essential cytokines [50]. It has been suggested that neutrophils are not required for fin regeneration [51], with their sustained presence inhibitory to the repair process [52]. However, neutrophils were shown to contribute to the structure of newly forming bone tissue in humans by secreting fibronectin into the extracellular matrix and providing a scaffold for stromal cells [53], suggesting their absence might exacerbate the effects on regeneration. Of potential relevance is myeloid-derived growth factor (MYDGF), which signals through STAT3 to promote osteogenesis [6], with the zebrafish MYDGF orthologue having roles in neutrophil migration in response to injury [54]. The inflammatory cytokine IL-6 was also shown to act via STAT3 [55], with the expression of the zebrafish *il6* gene shown to be elevated at later time points in an alternative Stat3 KO line [26]. However, basal levels were slightly decreased in both Stat3 KO and Stat3 ΔTAD mutants (Supplementary Figure S3), with its expression found to be induced in WT (2.55 ± 0.45 fold) and Stat3 KO (3.26 ± 0.54 fold) and Stat3 ΔTAD (2.13 ± 0.45 fold), with no significant differences between genotypes.

The Stat3 ΔTAD mutant lacks the entire transactivation domain, as well as the tyrosine residue phosphorylated via cytokine signaling to facilitate dimerization, and so abrogates canonical Stat3 function. Therefore, comparison with the Stat3 KO mutant can identify additional noncanonical roles. Like Stat3 KO mutants, Stat3 ΔTAD mutants displayed spinal deformities, reduced growth and survival, along with severely disrupted regeneration and innate immune cell recruitment following fin amputation, indicating that these effects are mediated by canonical signaling. However early bone development was largely unaffected in Stat3 ΔTAD mutants, while some regeneration (albeit defective) was observed. This suggests that early bone development and some aspects of regeneration are mediated by noncanonical Stat3 functions that are lost only in the Stat3 KO mutant. This is consistent with observations in humans where mutations in the IL-6 family signaling chain GP130 had less impact on bone phenotypes than STAT3 mutations [18]. Notably, STAT3 and STAT5 have been most frequently associated with noncanonical functions [9]. Interestingly, Stat3 ΔTAD mutants only showed impaired ossification of the ceratohyal bone, one of the few zebrafish bone structures that develops through endochondral ossification [21,56], suggesting this process is contingent on canonical signals.

## 4. Materials and Methods

### 4.1. Zebrafish Husbandry

Stat3 wildtype (WT), knockout (KO, *mdu35*), and transactivation domain truncation (ΔTAD, *mdu36*) zebrafish alleles were generated in-house (Sobah, M.L., et al., 2024 [28]) and crossed onto the Tg(*mpeg1.1*:GFP) [57] and Tg(*mpx*:GFP) [58] backgrounds obtained directly from their respective laboratories of origin. All lines were maintained using standard husbandry practices in a purpose-built aquarium (Tecniplast, West Chester, PA, USA) on a 14/10 h light/dark cycle with twice-daily feeding. Embryos were incubated at 28.5 °C in 1 × E3 water supplemented with 0.003% (*w*/*v*) 1-phenyl-2-thio-urea (PTU) (Sigma-Aldrich, Melbourne, Australia) to suppress pigmentation if required.

### 4.2. Experimental Setup

To minimize bias, all experiments were performed on offspring obtained from heterozygous in-crossing of each mutant, with quantitation performed in a blinded manner and genotyping performed postassay, as previously described [59].

### 4.3. Amputation Assay

Juvenile zebrafish were anesthetized using 15 μg/mL benzocaine in 1 × E3 water [60] and subsequently transferred to a glass slide where the distal portion of the caudal fin was amputated before returned to their original tanks. Fish were kept in normal conditions until imaging at the desired timepoints.

### 4.4. Alizarin Red Staining

Alizarin red staining of juvenile and adult zebrafish was performed as previously described [61]. Zebrafish were euthanized with 50 μg/mL benzocaine in 1 × E3 water and subsequently placed in bone-fixative solution (1.2 M formalin (Sigma-Aldrich, Australia), 0.1 M Triton X-100 (Promega, Fitchburg, WI, USA), 0.2 M potassium hydroxide (KOH)) for 24 h with rocking at 40 °C, then in enhancement solution (3.5 M ethylene glycol (Sigma-Aldrich, Australia), 0.1 M Triton X-100, 0.2 M KOH) for 24 h with rocking at 40 °C. Samples were then washed in distilled water for 5 min at room temperature (RT) before immersing into bone-staining medium (3.5 M ethylene glycol, 0.2 M KOH) for 15 min at RT. Next, samples were washed in bone-staining solution (0.2 M Alizarin red (Thermo-Fisher Scientific, Waltham, MA, USA), 3.5 M ethylene glycol, 0.2 M KOH) for 15 min with rocking at RT, followed by clearing solution (0.2 M Tween-20 (Thermo-Fisher Scientific, USA), 0.2 M KOH) for 24 h with rocking at 40 °C. Stained samples were stored in 4% PFA/PBS at 4 °C.

### 4.5. Calcein Staining

Calcein staining of zebrafish prior to their juvenile stages was performed as previously described [62]. Zebrafish were euthanized with 20 μg/mL benzocaine in 1 × E3 and immediately immersed in 0.5 M calcein (Sigma-Aldrich, Australia) in 1× Danieau buffer for 5 min with 6 dpf embryos and 15 min for 16 dpf juveniles, with three 15 min washes in 1 × E3 water before imaging.

### 4.6. Pulse Chase Staining

Regeneration following amputation was quantified at various timepoints, with a different batch of fish used for each respective timepoint. Zebrafish were euthanized in 50 μg/mL benzocaine and fully mineralized bone matrix was visualized using Alizarin red, and newly depositing bone matrix was visualized using calcein green [20]. Regenerating caudal fins were double-stained with Alizarin red (74 μM Alizarin red S, 5 mM HEPES (Promega, USA) and calcein (40 μM calcein green) made up in 1× Danieau, as previously described [63]. The region of amputation was imaged under UV excitation through the GFP and RFP filter for calcein and Alizarin red visualization, respectively.

### 4.7. Cartilage Staining

Embryos at 5 dpf were euthanized with benzocaine and fixed in 4% (*w*/*v*) paraformaldehyde (PFA) in PBS (PFA/PBS) for 30 min. The craniofacial cartilage of zebrafish embryos was stained with 0.015 mM Alcian blue (Sigma-Aldrich, Australia) and 200 mM MgCl_2_ (Sigma-Aldrich, Australia) in 70% (*v*/*v*) ethanol, as previously described [64]. Following staining, embryos were cleared with successive washes in 50% (*v*/*v*), 30% (*v*/*v*), and 10% (*v*/*v*) ethanol, followed by bleaching using 3% (*v*/*v*) H_2_O_2_.

### 4.8. Whole-Mount In Situ Hybridization

Embryos at desired timepoints were fixed in PFA/PBS prior to performing WISH with DIG-labeled antisense probes, as previously described [65].

### 4.9. Gene Expression Analysis

Total RNA was extracted from zebrafish embryos with an RNeasy Mini Kit (Qiagen, Germantown, MD, USA) and from juvenile zebrafish with TRIsure (Bioline, London, UK) according to the manufacturers’ protocols. This was subjected to quantitative real-time reverse-transcription PCR (qRT2-PCR), with data normalized relative to *actb* and fold-change calculated using the ΔCT method [66].

### 4.10. Imaging and Analysis

An Olympus (Shinjuku, Japan) MVX10 microscope with a DP74 camera was used for fluorescence and light microscopy. CellSens Dimension 1.6 software (Olympus, Japan) was used for image capture, with postprocessing performed with Adobe Lightroom and Photoshop 2024 (Adobe, San Jose, CA, USA).

### 4.11. Statistics

Statistical analysis was performed using Graphpad Prism v.8.0 (San Diego, CA, USA). Data were tested for homogeneity using a D’agostino–Pearson omnibus normality test to identify whether a parametric or nonparametric test was required. Depending on the variance, an ordinary one-way ANOVA with Tukey’s multiple comparisons or Kruskal–Wallis test was performed to compare the two different mutants with each other as well as with WT counterparts. Incidence of deformity and mortality was visualized using Kaplan–Meier plots with a log-rank (Mantel–Cox) test used to determine statistical significance.

## 5. Conclusions

Stat3 KO mutants exhibited growth defects and spinal abnormalities concomitant with impacts on bone but not cartilage formation, as reported in other studies. In addition, defective fin regeneration associated with abrogated infiltration of both macrophages and neutrophils was also identified in these mutants. Similar phenotypes were observed in Stat3 ΔTAD mutants, indicating canonical Stat3 functions were likely important, but the impacts on early bone formation and regeneration were weaker, suggesting these processes involve noncanonical Stat3 functions. This research highlights the myriad of complex roles played by Stat3 in both skeletal and immune cell biology; further research should focus on the interplay between them.

## Data Availability

The datasets generated or analyzed during this study are included in this published article (and its Supplementary Material file) or can be made available on request.

## Supplementary Materials

The supporting information can be downloaded at: https://www.mdpi.com/article/10.3390/ijms25010389/s1.
