# Supplementary material for: Contribution of Signal Transducer and Activator of Transcription 3 (STAT3) to Bone Development and Repair
Source: Int J Mol Sci. 2023 Dec 27;25(1):389. doi: 10.3390/ijms25010389 (PMC10778865; doi:10.3390/ijms25010389)
Supplement: Supplementary file 1 [file ijms-25-00389-s001.zip › ijms-2734676-supplementary.pdf]

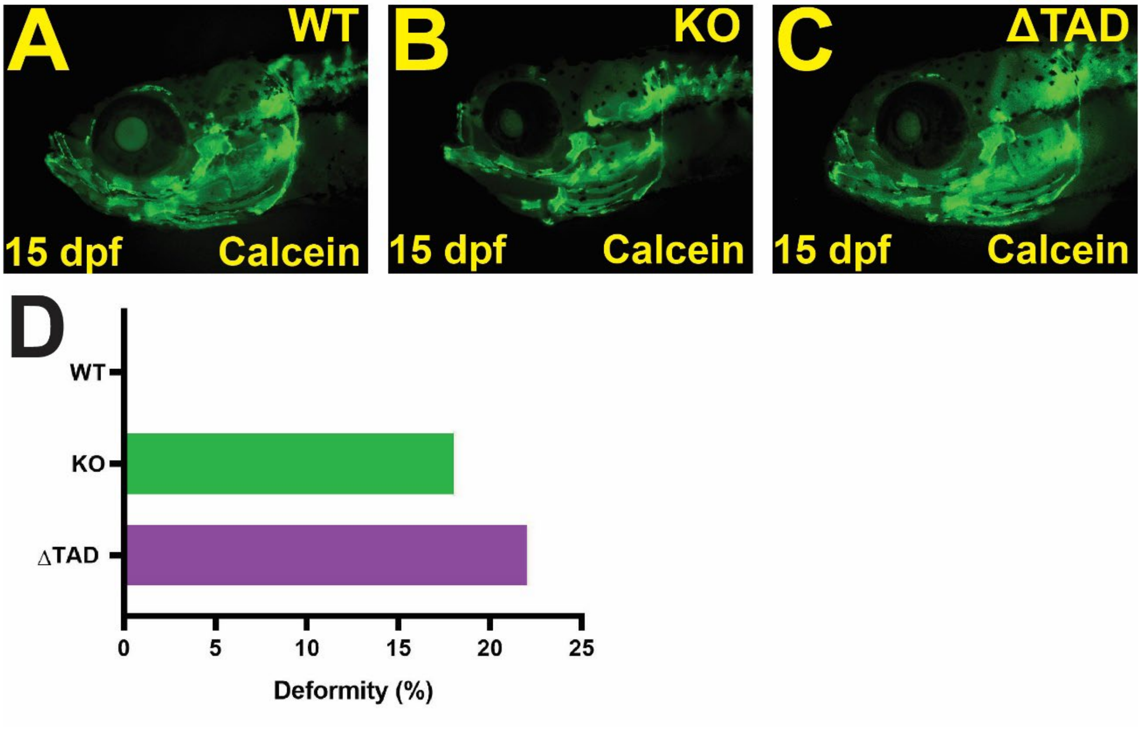


**Supplementary Figure S1.** Jaw deformity in Stat3 mutants. Calcein staining of Stat3 wildtype (WT: A), knockout (KO: B) and transactivation domain truncation (ΔTAD: C) mutants at 15 dpf, with quantification of jaw deformity frequency in each genotype (D).

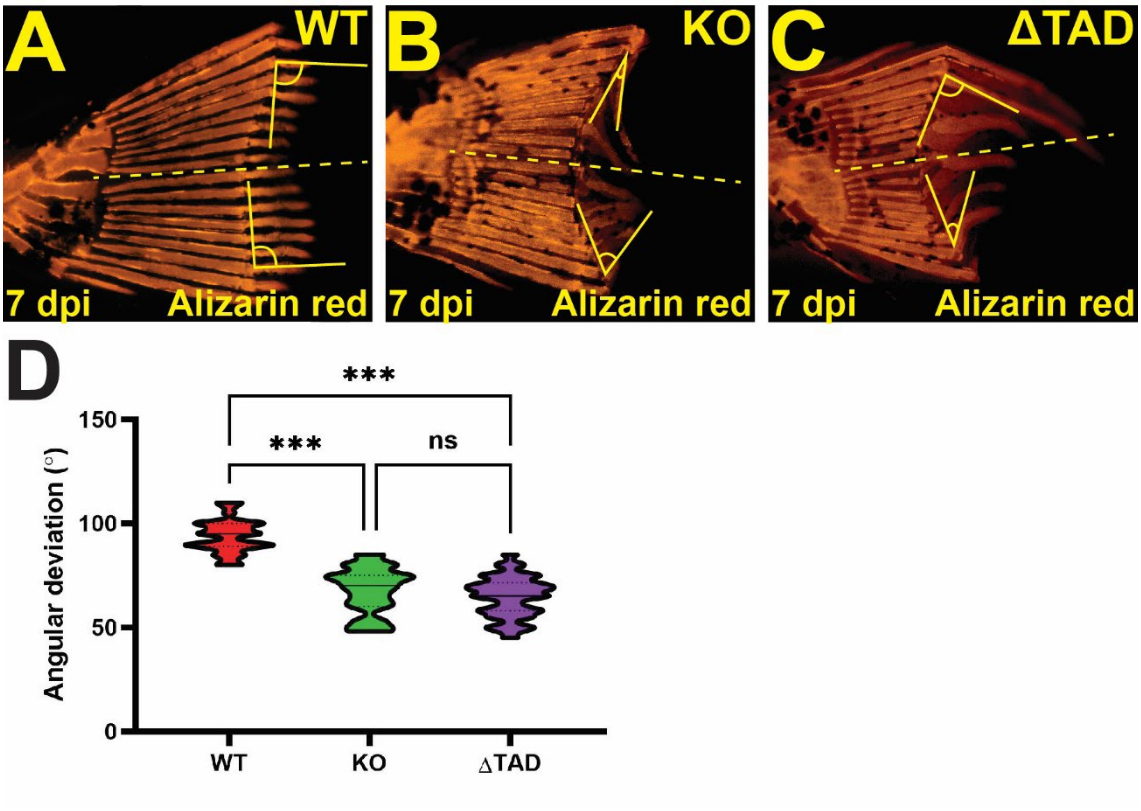


**Supplementary Figure S2.** Aberrant regeneration following caudal fin amputation in Stat3 mutants. Representative images of amputated caudal fin of juvenile Stat3 wildtype (WT: A), knockout (KO: B) and transactivation domain truncation (ΔTAD: C) zebrafish stained with Alizarin red at 7 dpi. (D) Quantitation of the angular deviation of regenerating fin rays presented as a Violin plot with median and range of angles presented, along with indication of statistical significance (*** $p < 0.001$, ns: not significant).

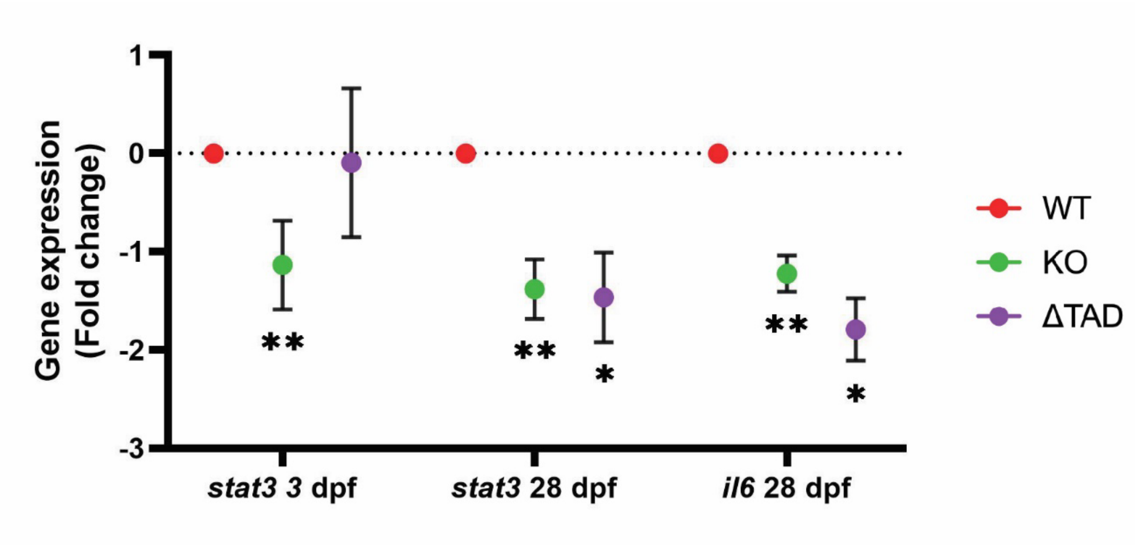


**Supplementary Figure S3.** Gene expression analysis in Stat3 mutants. Quantitation of expression for the indicated genes at the timepoints shown for Stat3 wildtype (WT), knockout (KO) and transactivation domain truncation (ΔTAD) mutants, presented as fold-change (log2) relative to WT, showing mean and SEM with statistical significance compared to WT (** $p < 0.01$, * $p < 0.05$).
